# Patient satisfaction with antiretroviral therapy service provided by pharmacists in Dembia district health institutions, Northwest Ethiopia

**DOI:** 10.1186/s12981-023-00533-z

**Published:** 2023-06-20

**Authors:** Tafere Mulaw Belete, Solomon Asmamaw Tadesse, Kidist Atnafu, Minilik Kelemu, Assefa Belay Asrie

**Affiliations:** Department of Pharmacology, College of Medicine and Health Sciences, University of Gondar, P.O.Box 196, Gondar, Ethiopia

**Keywords:** Antiretroviral therapy, HIV/AIDS, Health center, Patient satisfaction

## Abstract

**Background:**

The patients’ perception of the health service is a vital tool for measuring health service quality. Besides, Patient satisfaction is an essential feature in assessing the quality of health services. Health institution leaders are considering quantifiable patient satisfaction data as a means to evaluate the health care service.

**Method:**

An institution-based cross-sectional study was employed from 21/8/2022 to 21/9/2022 among 308 patients attending ART pharmacy services in three health institutions of Dembia distinct. Data were collected by using a questionnaire and reviewing medical charts. Results were calculated and presented in the form of texts, tables, and graphs. Variables with a *p*-value of 0.05 were considered significant determinants of patient satisfaction.

**Result:**

A total of 308 HIV patients were recruited with a response rate of 100%. The overall prevalence of satisfaction among respondents was 231(75%). Being unable to read and write [1.21(AOR = 1.07–4.31)] and patient age greater than 48 years 1.9(0.73–2.59) were significantly associated with the level of patient satisfaction. Among the participants 66.9% were satisfied with clear and organized service, and 76% were satisfied with the convenience of a private counseling room.

**Conclusion:**

The general patient satisfaction at the antiretroviral therapy clinic did not achieve the national target of 85% satisfaction with significant differences among health centers. Being educated to a higher level, absence of signs and directions to ART clinics, and not having the opportunity to ask questions were the factors influencing patient satisfaction with ART service.

## Introduction

HIV/AIDS is the most severe and critical public health problem, which has claimed 40.1 million [33.6–48.6 million] lives since the beginning of the epidemic. At the end of 2021, more than 38.4 million [33.9–43.8 million] people are living with HIV/AIDS, of which 28.7 million people were accessing ART. Sub-Saharan African countries, including Ethiopia, carry the most of the burden accounting for 75% of AIDS-related deaths. Antiretroviral therapy (ART) is a great public health success that improved the survival of HIV-infected people [1, 2]. In Ethiopia, HIV/AIDS infection has claimed 1.3 million lives since the very beginning of the epidemic. The prevalence of HIV/AIDS in Ethiopia was 1.16%, despite a significant regional difference of 1.2% in the Amhara region and 4.2% in the Gambella region [3]. Because of the non-affordability of ART by most HIV-infected individuals in Ethiopia, the Ministry of Health launched free ART access in January 2005. Since then, in Ethiopia, about 738,976 people are living with HIV, and all of them need ART, but only 426,000 people were accessing ART [4]. It is still difficult to accomplish universal access to high-quality HIV/AIDS health services in developing countries with prevalent HIV epidemics [5].

The quality of health care is a global issue that needs a rapid transformation to meet client demand. Most of the time, health service quality depends on health professional standards. But, the patient’s perception of the health service is a vital tool for measuring health service quality. Besides, Patient satisfaction (PS) is an essential feature in assessing the quality of health services. Health institution leaders are considering quantifiable patient satisfaction data as a means to evaluate the health care service [6]. PS is a component of the health system that helps to evaluate the quality of the health system. PS assesses the relationship of patient-physician, the health institution environment, and the accessibility of the health service, especially for patients with chronic disease follow-up [7]. The quality of the service affects patients’ adherence to drugs. Several studies revealed that more satisfied patients have greater medication adherence, keep their schedules, trust the health professional, and possess better health outcomes. Patients are satisfied when their interests and expectations are found in the health facilities [7, 8].

PS among HIV/AIDS leads to better adherence to regimen and management success, such as better survival and fewer opportunistic infections. PS can be affected by the treatment process and outcome [8, 9]. Dissatisfaction may reduce the patient’s willingness to adhere to the ART treatment [9]. The socio-demographic characteristics, health situations of the patient, and health institution latrine hygiene were among the factors that affect PS [10]. Even if several activities are done to achieve PS, still, it is hard to fulfill universal PS, particularly in sub-Saharan African countries, including Ethiopia [10]. Pharmacists are drug experts who deliver information about a drug that facilitates safe and cost-effective use of the drug. Pharmacists communicate with patients during counseling and dispensing [11]. Patients’ attitude to pharmaceutical services is one of the challenges during chronic disease treatment. It affects the patient’s attitude to drug safety and health care service and minimizes the acceptability of drugs and adherence [12]. A study done in Benin showed 45.1% of the participant agreed with the key role of pharmacists in patient care with chronic illness [13]. Another study in Nigeria showed that the perception of the participant to the role of pharmacist in pharmaceutical care was 86% [14].

Understanding patients’ views of pharmaceutical services as an important component of the health service may help to know the gap and inform improvements. Even though many studies have shown the difficulty of HIV/AIDS prevention and control programs, data about the amount and patterns of patients’ expectation and PS with the pharmaceutical services in the health facilities which are the component of health care service are still scarce in Ethiopia [15, 16]. Therefore, this study aimed to evaluate the level and associated factors of satisfaction with health service delivered by pharmacists in Dembia district health institutions, in Northwest Ethiopia. Finally, our study facilitates to increase in pharmaceutical services quality at Dembia district health facilities.

## Methods

### Study design, area, and period

An institution-based cross-sectional survey using an interviewer-administered questionnaire was used to evaluate the extent of satisfaction and associated factors among HIV/AIDS patients who get services in Kola Diba health center, Kola Deba primary hospital, and Chuahit health center ART pharmacies. Dembia District is found in central Gondar, Amhara region, 730 km north of Addis Ababa. Dembia district is bordered on the southwest by Takusa, on the south by Lake Tana, on the north by Lay Armachiho, on the west by Chilga, and east by Gondar Zuria. In 2017, the Dembia district has a total population of 326,686 with an area of 1,261.96 square kilometers. There are 10 Health Centers and 40 Health Posts in the district. The study was conducted from June 10 to August 10, 2022.

### Source population and study population

The source population was all adult HIV/AIDS patients who had ART follow-ups and attended the ART clinic at each health institution were considered as source population. The study population was all adult clients already receiving ART and has follow-up at ART clinic from June 10 to August 10, 2022.

### Inclusion and exclusion criteria

Inclusion criteria include all adult patients (18 and greater than 18 years) living with HIV/AIDS and willing to participate in the study, can communicate in the local language i.e. Amharic language, and finish the interview. Exclusion criteria, including psychiatric patients that could not understand reality, and patients with emergency medical conditions were not included as study participants. Patients who are critically ill or have mental diseases were also excluded.

### Sample size determination and sampling technique

The sample size was calculated by applying single population proportion formula by taking PS level P = 0.526 (percentage of PS in pharmacy service from the previous study in Ethiopia) [15], Z = the required degree of accuracy at 95% confidence interval [CI], (Z = 1.96), considering marginal error (d) of 5% (d = 0.05), Where n = required number of sample size,

n = z2 p (1-p) / d2 = (1.96)² 0.526(1-0.415)/ (0.05)² =373.05 ≈ 373.

A correction formula was used to adjust the sample size because all eligible clients receiving ART drugs during the study period (in the year 2022) were nt = 1124 which is less than 10,000 source populations. A correction formula.

$${n_f} = \frac{{{n_i}}}{{1 + {n_i}/{n_t}}},$$

Where *n*_i_ is the initial sample size (the calculate sample size); n_f_ is the final required sample size; *n*_t_ is the total number of eligible clients receiving ART drugs during the study period. Hence, the sample size was calculated at a total of source population n_t_ = 1124 and n = 384 and n_f_ = 280. To get a more representative sample of the target population, the authors’ added a 10% non-response rate or incomplete data; the final sample size (*n*_f_) became 308 adults on ART.

The study participants were selected by proportionally assigned (population proportion method) to individual health facilities based on the number of patients on follow-ups on the ART clinic. The required numbers of respondents were selected from individual health facilities by using a systematic random sampling technique.

A total number of patients who visited ART clinic from June 10 to August 10, 2022 were 1124. The sampling interval was established by dividing the total number of patients that visited the ART clinic with the sample size that can be collected in each health institution. Therefore, the sampling interval was calculated for each health institution, and the first participant selected by simple random sampling. Thereafter, based on the sampling interval participants come to the health institutions selected until the required sample number was obtained. Accordingly, 172, 28, and 108 ART clinic visitors were systematically selected from Kola Deba health center, Kola Deba primary hospital, and Chuahit health center.

### Study variables

Patients’ demographic characteristics (sex, religion, age, residence, monthly income, level of education, occupation), the clinical status of the patient, the distance of health facility from the residence area, ART regimen, availability of drugs, adherence status, and communication level were the independent variable. The patient’s satisfaction level was the dependent variable.

### Data collection tool and procedure

Data collection was performed by using structured questionnaires by interviews. The questionnaire was adapted from similar literature and modifications were made to fit our study objective. The questionnaire was first prepared in English and then translated into Amharic. The questioner posse’s socio-demographics character, the health facilities’ setting, health professional communication, and health service accessibility to evaluate PS. Pre-tested was done on 5% of the total sample size in a similar setting to the study area to keep the consistency and validity of the tool. A short training was delivered to supervisors and data collectors on how to collect data and check questionnaire completeness. Data on socio-demographic properties such as age, sex, and distance from the health facilities were collected via interview, but clinical data such as ART regimen, and viral load were extracted from the medical charts of the patient.

### Measurements and operational definitions

Participants rated their satisfaction levels for the services, using a five-point Likert scale with five items (1-very dissatisfied, 2-dissatisfied, 3-neutral, 4-satisfied, and 5-very satisfied). These scales were adopted from previous studies because there is no standardized satisfaction measurement tool. The Cronbach’s alpha test was used to evaluate the reliability or consistency of the PS assessment tool. Cronbach’s Alpha range is between 0 and 1, with greater values showing better questionnaire consistency. Satisfaction: the fulfillment of patients’ ideal needs, desires, or expectations about the real healthcare service. Satisfied: PS scores greater than 60%. Dissatisfied: PS scores below 60%. Good adherent: if the patients’ adherence is more than (95% or < 3 doses missed per month). Fair adherent: (85–94% or 4–8 doses per month). (< 85% or > 9 doses missed per month) [17, 18].

### Data processing and analysis

After data collection, the questionnaires were checked for clarity and completeness. The data were entered into and analyzed using SPSS version 25. Bivariate analysis was done and variables with a p-value less than 0.05 were included in regressions analysis to control the confounder factor and identify the significant predictor of outcome variables. Descriptive analysis was performed for the study variables and frequency distribution tables were used to describe most of the study findings.

### Ethical consideration

Ethical clearance was obtained from the review committee of the School of Pharmacy, university of Gondar with approval no of SOP/257/2022. Necessary permissions were gained from the health facilities. The study was done according to the criteria set by the Declaration of Helsinki. The respondents were informed about the objective of the study and their verbal consent to participate was obtained before collecting data. Data were collected after their informed consent is obtained and confidentiality of the data was maintained.

## Result

### Participant characteristics

Out of 308 respondents interviewed, the majority of respondents were females 63.6%, and from the urban area (55.8%)., those in the age group of 37–47 years accounted for the highest proportion 157 (50.9%) followed by the age group of < 37 years (35.4%). Among the participants, 97 (31.3%) were farmers and 71(22.9%) were government employees. Among the participant 269 (87%) monthly incomes was greater 2400 birr. The result displayed that most participants had an educational level of unable to read and write was 174 (41.5%). The majority of the respondents were from Kola Deba health center 167 (54.22%), followed by 112 (36.36%) from Chuahit health center, and the remaining 29(9.4%) from Kola Deba primary hospital as presented in Table 1.

**Table 1:** Socio-demographic characteristics of respondents at ART service at Dembia Woreda health center, Northwest Ethiopia

Variables	Category	Frequency	Percent
Age	< 37	109	35.4%
	38–47	157	50.9%
	> 48	42	13.6%
Gender	Female	196	63.6%
	Male	112	38.5%
Educational level	Unable to read and write	127	41.2%
	Able to Read and write	74	23.9%
	Primary school (1–8)	20	6.4%
	Secondary school (9–12)	35	11.5%
	College and above	52	16.7%
residence	Urban	172	55.8%
	Rural	136	44.2%
Marital status	Single	29	9.5%
	Married	145	47.0%
	Divorced	56	18.1%
	Widowed	78	25.3%
Occupation	Housewife	32	10.3%
	Other(farmer)	96	31.3%
	Student	12	3.8%
	Government employee	71	22.9%
	Private worker	26	8.4%
	Daily labor	40	12.9%
	Merchant	27	8.6%
	Unemployed	6	1.9%
Income	< 1200	30	9.7%
	1201–2400	40	12.98%
	> 2400	238	72.27%

### Clinical characteristics of participants

The average percent of duration in which HIV//AIDS patients receive ART was 68.36 (± 32) months, and about 63.0% of the respondents were on ART for greater than 58 months. Almost all patients receive TLD (Tenofovir disoproxil, lamivudine, dolutegravir) ART regimen, and most of them (96.2%) had a recent viral load below 1000 copies/ml. Three hundred participants possess good adherence. About 13.2% of the respondents had opportunistic infections at the time of data collection. About 76.6% of the respondents disclose their serological status and it took less than 30 min to reach the health center for about 306 (73.0%) of the study participants as shown in Table 2.

**Table 2:** Clinical Characteristics of Patients at Dembia Woreda Health Institutions, Northwest Ethiopia

Variable	Category	Frequency	Percent
Duration of treatment	< 60 months	145	47.1%
	>_60 months	163	52.9%
Recent viral load	< 1000 copies/ml	296	96.2%
	>_1000 copies/ml	12	3.8%
ART regimen	1. AZT/3TC/NEV	3	0.5%
	2. TDF/3TC/EFA	4	1.2%
	TLD	301	98.3%
Adherence	Good	300	97.6%
	Fair	7	2.1%
	Poor	1	0.2%
Perceived HIV disclosure	Yes	236	76.6%
	No	72	23.4%
Frequency of visit	Every month	10	3.3%
	Every three month	99	32.0%
	Every six month	199	64.7%
Time taken to reach hospital	< 30 min.	225	73.0%
	30–60 min.	61	19.8%
	> 60 min.	22	7.2%
Opportunistic infections	yes	41	13.3
	No	267	86.7

### Clients’ satisfaction with ART pharmacy and latrine location

This study showed that 75% of the participants replied satisfied with the ART pharmacy location. Most participants responded highly satisfied (66.9%) with the provision of clear and organized service, and 76% were satisfied with the convenience of a private counseling room. Several patients were less satisfied with latrines access (55.5%) and comfort (35.7%) at the health facilities as presented in Table 3.

**Table 3:** Level of satisfaction of clients at ART pharmacy and latrine location

Questions	1 No (%)	2 No (%)	3 No (%)	4 No (%)	5 No (%)
Convenience of ART pharmacy location (including sign for directions)	24(7.8)	40(13)	13(3.9)	206 (66.9)	25(8.1)
Comfort of waiting area	28(9.1)	62(20.1)	52(16.9)	163(52.9)	3(0.97)
cleanness and tidiness of ART pharmacy	24(7.8)	59(19.2)	34(11.1)	176(57.1)	15(5.2)
Provision of clear and organized service	24(7.8)	52(16.9)	37(12)	182(59.1)	24(7.8)
convenience of private counseling room	10(3.3)	24(7.8)	40(13)	206 (66.9)	28(9.1)
Comfort ability of seating chair	10(3.3)	71(23.1)	10(3.3)	204(66.23)	13(4.2)
Waiting time until getting the service	3(0.97)	26(8.45)	17(5.52)	238(77.27)	24(7.8)
Availability and accessibility of latrine	52(16.9)	43(13.9)	34(11.1)	168(54.54)	3(0.97)
Cleanness and comfortability of latrine	80(26)	87(28.3)	43(13.9)	93(30.2)	16(5.2)

**Key**: Response 1-very dissatisfied, 2- dissatisfied, 3- neutral, 4 – satisfied, 5 - very satisfied

### Satisfaction of patients with ART pharmacy services

**Fig. 1:**
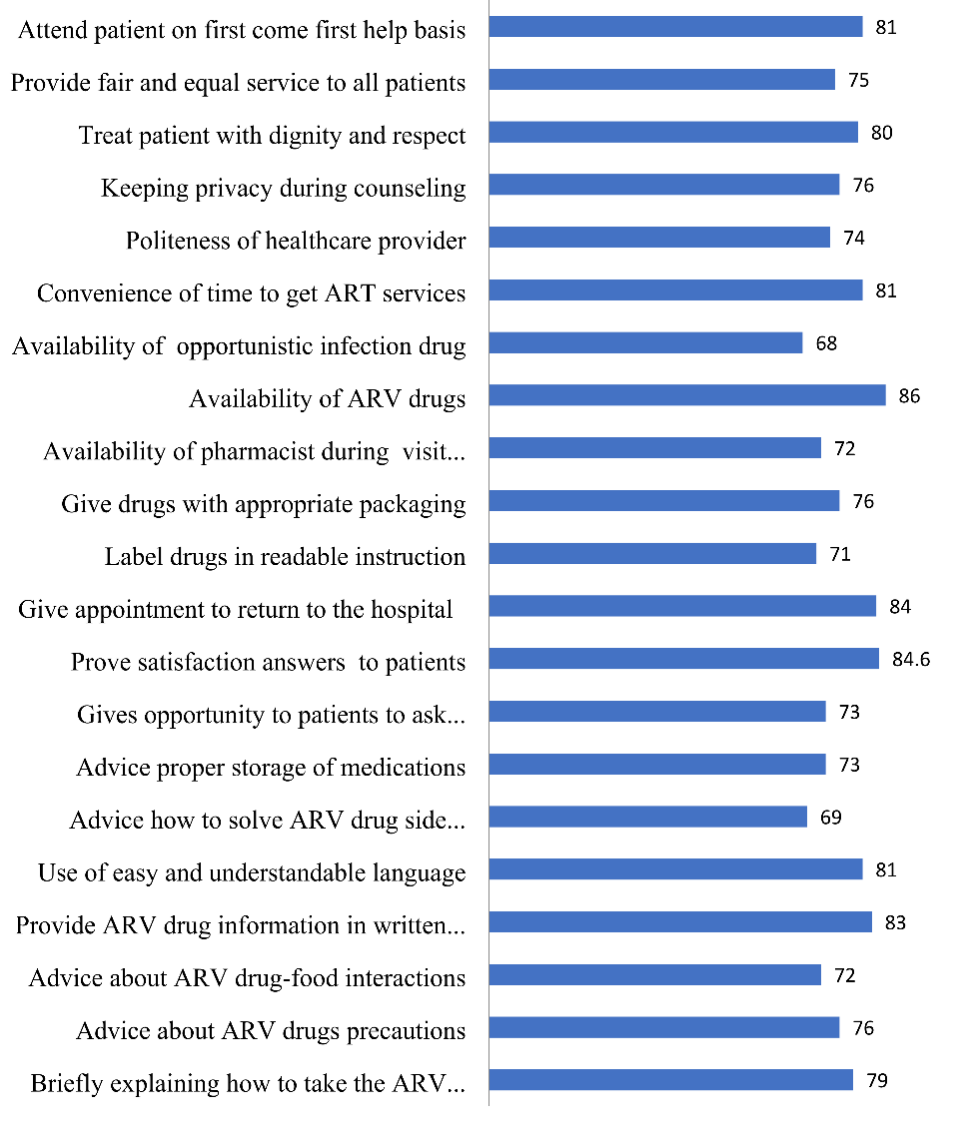
Satisfaction of HIV/AIDS patients with ART pharmacy services

This study showed that satisfaction with patients’ ART service was 76.95% [(95% CI: 78.7, 83.1)]. PS with the main items, including briefly explaining how to take the ARV drugs 243 (79%), advice on how to solve ARV drug side effects 212 (69%), labeling medicines in readable and understandable instruction 219 (71%), advice about proper storage of medications 225 (73.1%), keeping privacy during counseling services 234 (76%), availability of pharmacist during visit 222 (72.1%) and giving medicines with appropriate packaging 234 (76%) were satisfied respectively. Whereas the availability of opportunistic infection drugs 210 (68.2%) of the patients were satisfied as shown in Fig. 1.

### Associated factors that affect PS on ART services

This study showed that the general PS at ART pharmacy service was 76.95%. [(95% CI: 72.7, 81.1)]. The individual health facilities PS found 29 (75%) in Kola Deba primary hospital and 167 (80.23%) in Kola Deba health center. For patients with ages 38 to 47 years and greater than 48 years, the odds of satisfaction were 1.37 (AOR = 5.91;95% CI:3.36–10.32) and 1.9 (AOR = 2.67;95% CI: 1.38–5.12) times higher, respectively, as compared patients aged less than 37 years. For Patients with primary school [1–8] education, the odds of satisfaction were 1.22(AOR = 1.15:95% CI: 0.87–2.31) times higher compared to patients with college and above educational level. Patients who lived in urban areas were 1.05 times more satisfied (AOR = 1.27: 95% CI: 1.01–2.07) than those who lived in rural residential areas. Compared to patients who had to follow up the satisfaction of patient at the Dembia Woreda health facilities, the odds of satisfaction at Kola Deba HC and Kola Deba primary hospital were 1.67 (AOR = 2.67; 95%CI: 1.46 2.47) and 0.58 (AOR = 4.4; 95%CI: 1.72, 11.31) times as shown in Table 4.

**Table 4:** Factors affecting PS on antiretroviral therapy services at Dembia Woreda health facilities, Northwest Ethiopia

Variable	Category	satisfaction		COR 22	AOR	P-value
		yes	no			
Age group	<=37	79	30	1	1	0.91
	38–47	123	34	1.37(0.41–2.09)	1.04(0.51–2.03)	0.93
	> 48	35	7	1.9(0.73–2.59)	1.28(0.33–2.39)	0.02
Marital status	Single	22	7	0.86(0.47–2.02)	1.07(0.33–3.43)	0.91
	Married	110	35	0.87(0.63–1.61)	1.03(0.53–1.53)	0.93
	Divorced	44	12	1.04(0.76–2.44)	1.12(0.85–2.38)	0.66
	Widowed	61	17	1	1	0.93
Educational level	Unable to read and write	101	30	1.03(0.51–2.09)	2.36(0.77–2.6)	0.03
	Able to Read and write	60	14	1.21(0.84–1.72)	1.49(0.59–2.74)	0.06
	Primary school (1–8)	16	4	1.22(0.73–1.67)	0.75(1.07–2.31)	0.05
	Secondary school (9–12)	26	9	0.88(0.63–2.07)	1.03(0.76–2.77)	0.17
	College and above	46	14	1	1	1
Monthly income	< 1200	22	7	1	1	1
	1201–2400	30	10	0.96(0.52–2.56)	0.64(0.98–4.2)	0.65
	> 2400	215	54	1.27(0.53–1.59)	0.82(0.46–1.66)	0.27
Residence	Urban	133	39	1.05(0.53–1.59)	1.27(0.53–1.59)	
	Rural	104	32	1	1	
Study site	Kola Deba HC	137	30	1.67(0.40–0.91)	0.67(0.73–2.54)	0.33
	Kola deba PH	18	11	0.59(0.36–1.61)	0.93(0.65–2.39)	0.74
	Chuahit HC	82	30	1	1	1

## Discussion

PS in the pharmaceutical services helps to measure the outcome of the healthcare, and improve the quality of the pharmacy service. Therefore, this study determined the magnitude of PS and associated factors with ART pharmacy services. In our study PS toward services provided by pharmacy professionals was 76.95%. The age of the participants, marital status, educational level, and residence were factors that affect PS with ART pharmacy services. The variations in PS among health facilities may relate to health facilities setups differences, experiences, and resource availability in the health facilities. PS level in our study was similar to the study undertaken at a public hospital in Ghana (75%) [19]. The possible reason for this result being in line with the previous study may be related to the similarities of some determinant socio-demographic and clinical characteristics and similarities of tools used to assess the level of satisfaction. All of the mentioned factors differentiate from one institution to another.

PS measurement has a significant implication for service providers to know the limitation and improve the quality of service. However, this result was lower than the result in South Africa 86% [20], Addis Ababa hospitals (85.5%) [21], and Tigray region (89.6%) [22]. The reason may be associated with socio-demography, awareness of clients about pharmacy services, study setting, and service site. In addition, hospitals have a better chance to get drugs and pharmacists that deliver patient-assumed service than health centers. But, this study’s result was greater than the result at the University of Gondar Hospital (54.7%) [15] and Tanzania (65.2%). The reason may be the quality of service, and a study undertaken at the University of Gondar Hospital included private health facilities that may affect PS.

Among the domains used to assess PS, the availability of ARV medication (86%) showed high PS rating scores. This might be explained by having a free supply of ARV drugs will definitely take their level of satisfaction to a higher level. But, this study was lower than the former studies done in Jimma 89.5% [11]. The variation may be related to the presence of equipped pharmacy service (drug and medical equipment). As a result, the present finding proposes ARV medication availability is a core pharmacy service to be satisfied [23].

The domains such as availability, cleanness, and comfort of latrines received lower scores. The reason may be poor latrine hygiene in health institutions may increase the incidence of nosocomial infections that may affect patients’ safety and satisfaction.

The other domains were the comfort of the seating chair. About 63.7% of respondents in this study were satisfied with the comfort of a seating chair. The possible reason for this might be the availability of organized ART care structures in health facilities. This study revealed that higher PS (84.6%) was reported on o health professionals’ answers to patients, providing ARV drug information in written form (83%) and availability of ARV drugs 86%. This study was similar to studies done by Mekonnen et al. on eastern Ethiopia hospitals [7]. Our study displayed patients greater than 48 years old showed increased satisfaction with ART services than younger ones. This may be related to older patients who are usually stable in psychology and better satisfied with services get at the health institution.

For Patients with primary school [1–8] educational level, the odds of satisfaction were 1.22(AOR = 1.15:95% CI: 0.87–2.31) times higher compared to patients with college and above educational level. This result is similar to other research that was conducted in Hosanna and eastern Ethiopia [7]. The reason in line with our study may be because patients with primary education may have less expectation of ART services due to this reason may be better satisfied than a participant who completed college and above education level.

This study lacks a qualitative component that is vital to evaluate the insight and beliefs of the patient. The study was cross-sectional, so it is hard to show the prevailing problems at a given point of time. Besides, the satisfaction measurement tool is not standardized and may introduce bias. The dichotomization Likert scale may disrupt the data nature.

## Conclusion

In this study, general PS with ART service was less than the national target of 85% with a significant difference among health institutions. This study showed a significant improvement needed in the quality of health service and accessibility of recourses in the health centers. The low satisfaction parameters should be further studied to solve the problems. The health facilities must apply good dispensing practices and provide continuing professional development to pharmacists to improve PS.

## Data Availability

All the data generated during this study included within the article.

## Abbreviations

ART: Antiretroviral therapy
PS: Patient satisfaction
